# Immunotherapies for Neurodegenerative Diseases

**DOI:** 10.3389/fneur.2021.654739

**Published:** 2021-06-07

**Authors:** Ibrahim Mortada, Raymond Farah, Sanaa Nabha, David M. Ojcius, Youssef Fares, Wassim Y. Almawi, Najwane Said Sadier

**Affiliations:** ^1^Neuroscience Research Center, Faculty of Medical Sciences, Lebanese University, Beirut, Lebanon; ^2^Department of Biomedical Sciences, University of the Pacific, Arthur Dugoni School of Dentistry, San Francisco, CA, United States; ^3^College of Health Sciences, Abu Dhabi University, Abu Dhabi, United Arab Emirates

**Keywords:** neurodegenerative diseases, neuropathology, neuroinflammation, anti-inflammatory agents, immunotherapy, active immunization, passive immunization, vaccination

## Abstract

The current treatments for neurodegenerative diseases are mostly symptomatic without affecting the underlying cause of disease. Emerging evidence supports a potential role for immunotherapy in the management of disease progression. Numerous reports raise the exciting prospect that either the immune system or its derivative components could be harnessed to fight the misfolded and aggregated proteins that accumulate in several neurodegenerative diseases. Passive and active vaccinations using monoclonal antibodies and specific antigens that induce adaptive immune responses are currently under evaluation for their potential use in the development of immunotherapies. In this review, we aim to shed light on prominent immunotherapeutic strategies being developed to fight neuroinflammation-induced neurodegeneration, with a focus on innovative immunotherapies such as vaccination therapy.

## Introduction

Inflammation of nervous tissue, termed neuroinflammation, occurs in response to diverse cues, such as infection, traumatic brain injury, toxic metabolites, or autoimmunity. Neuroinflammation, which is an important process for maintaining healthy central nervous system (CNS) function following injuries such as physical trauma and infections, is highly regulated due to the lack of regenerative ability of post-mitotic cells of the nervous system. It also constitutes a major component of many neurodegenerative (1) and psychiatric disorders (2).

Inflammatory responses in the CNS are induced by microglia, the resident innate immune cells, and further exacerbated by reactive astrocytes and infiltrating leukocytes (3). They are usually brief and followed by the recruitment of other immune cells to the affected area, clearance of the insulting agent, and tissue repair or scarring, leading to immune resolution. These events together constitute acute neuroinflammation, as opposed to chronic neuroinflammation, which may persist for decades. The majority of neurodegenerative disorders display low-grade chronic neuroinflammation, which can result in collateral damage worse than the original insult (4). In addition to disrupting the neurocircuitry, these conditions ultimately may cause permanent neuronal damage and brain atrophy, which are thought to result from the sustained release of cytotoxic factors by activated microglia, astrocytes and other immune cells, leading to neurodegeneration (4).

Neurodegenerative diseases are characterized by a progressive loss of neurons in several areas of the CNS; and are associated with cognitive, psychiatric, and motor deficits due to atrophy of the affected regions (5). Together, neurodegenerative diseases exert a major global disease burden, with dementia being a public health challenge in many developed countries. As aging is a strong risk factor for the most common neurodegenerative conditions, the global economic and social impact of these diseases on healthcare systems will likely continue to surge significantly in the coming decades due to increasingly aging populations and longer life spans (6). It has been projected that by the year 2050, the population of individuals over the age of 60 will rise from 901 million in 2015 to 2.1 billion people worldwide (7). The increased life expectancy will be accompanied by higher age-related diseases, with the elderly expected to spend most of their later years in ill-health. In fact, a main cause of disability in the elderly is dementia, affecting 44 million people globally, and expected to surpass 135 million people by the year 2050 (8). Over 36 million people worldwide are diagnosed with Alzheimer's disease (AD) or Parkinson's disease (PD), the two most common neurodegenerative disorders. The absence of effective disease-modifying treatments and the failure of most clinical trials for new therapies highlight the need to identify new therapeutic targets to halt disease progression. An important challenge in the developing treatment strategies for most progressive neurodegenerative diseases is their multi-factorial etiology and diverse disease course (9–11). For the most common neurodegenerative diseases, such as AD, PD, amyotrophic lateral sclerosis (ALS), and Huntington's disease (HD), the causes of disease occurrence and progression are not fully known. Moreover, the disease course and severity varies significantly among patients, which complicates the challenge of efficient therapeutic interventions.

Common pathological mechanisms identified in most progressive neurodegenerative diseases involve neurotoxic protein misfolding, oxidative stress, and proteasomal impairment (4, 12). Increasing evidence suggests the presence of causal mechanistic links between toxic misfolded protein assemblies and neurodegeneration. Atypical protein aggregates are currently considered a main feature of most neurodegenerative disorders including PD, ALS, and HD, although their pathological significance is still debated (13).

Most articles in the literature describing immunotherapies for neurodegenerative diseases have typically focused on auto-immune neurodegenerative disorders such as multiple sclerosis (14). Until recently, little was known about immunotherapeutic interventions targeting aging-associated as well as other non-auto-immune neurodegenerative diseases. In this review, we therefore highlight recent immunotherapeutic strategies being developed to treat neuroinflammation-induced neurodegeneration, with a focus on immunotherapies.

## Pathogenesis Of Neuroinflammation

### Role of Microglia and Astrocytes

Microglia are resident macrophages in the brain and spinal cord and the primary immunocompetent cells in the CNS (15). Microglia constitute ~15% of the total cells in the brain, and their numbers and densities change depending on the brain region (16, 17). In the normal adult brain, microglia usually exist under different phenotypic states depending on the brain activity, plasticity, and response to challenges throughout life. Cutting-edge fate mapping and imaging techniques, along with RNA-seq expression profiling and other complementary technologies, have helped to decipher the origins, functions, and different phenotypes adopted by microglial cells (18, 19). During CNS surveillance, microglia dynamically remodel the structure of their processes and shift from a “surveying” ramified state to reactive amoeboid form in response to disturbances in homeostasis. Cells depart from the surveillance mode and acquire a reactive profile tailored to cope adequately with each specific trigger. This includes chemotactic reorientations, up-regulation of several cell surface proteins, release of numerous secreted factors and other transcriptional adjustments, which can occur in minutes or within a few hours post activation (20). With adjacent microglia surveying the brain parenchyma, studies estimate that the entire neuronal network of the brain can be scanned within hours (20–22). These cells are critical for proper brain development and maintenance of brain homeostasis throughout the life span, and their activation processes seem to be more diverse and dynamic than previously anticipated at all cellular levels (23). Based on the rapidly growing literature and the remaining gaps in microglial incontrovertible status within the CNS, there is an urgent need to create an updated microglial terminology that takes into consideration previous and newly discovered knowledge, including transcriptomic and proteomic profiles in both brain health and disease.

Astrocytes are the most abundant glial cell type in the CNS, providing mechanical and metabolic support to neurons, and helping in the regulation of critical biochemical activities including maintenance of the neural network, ionic and extracellular space volume homeostasis, synaptic plasticity, and blood flow (24). In response to a pathologic insult, astrocytes modify their morphological and functional state and become activated, which can be beneficial (radial glial-like astrocytes) or detrimental (reactive astrocytes) (24). Upon activation, astrocytes promote a pro-inflammatory environment by releasing mediators, such as IL-6 and TNFα. They can also recruit peripheral and CNS immune cells to the site of neuronal injury and degeneration (25). Furthermore, because astrocytes are the gatekeepers of the blood-brain barrier (BBB), they are associated with its pathological breakdown, which occurs during progressive neurodegeneration. The breakdown of BBB is thought to facilitate the entry of peripheral immune cells and blood components, which can exacerbate CNS neuroinflammation and neurodegeneration (26, 27). Nonetheless, the main functions of astrocyte activation and inflammatory responses aim to limit CNS damage and tissue remodeling, and to enhance neuronal recovery. Some studies have highlighted the role of astrocytic degeneration and atrophy in the early stages of neurodegenerative diseases, which may have implications for neuronal loss and disease progression (26).

## Mechanisms Of Neuroinflammation Mediated Neurodegeneration

The main feature of neuroinflammation in the CNS is the presence of persistently-activated primary immunocompetent glial cells, microglia, and astrocytes, at the areas of neurodegeneration. The progressive neuronal loss is typically accompanied by the presence of hypertrophic microglia and dystrophic astrocytes. The primary functions of this reactive gliosis were thought to be phagocytic clearance and immune surveillance in the case of activated microglia, and neurotrophic support for astrocytes. This holds true in most cases of acute brain injury or infection, where neuroinflammatory responses are regulated and usually subside when the infection clears or homeostasis is restored at the location of injury. This transient activation of microglia mediates a microenvironment which can promote neuronal survival and recovery of CNS homeostasis (28–30). Acute and regulated microglial activation drives anti-inflammatory and neurotrophic responses, and protects neuronal populations from excitotoxic injury (31, 32). The pro-homeostatic effects of microglia is also evidenced by *in vivo* studies of microglia depletion and replenishment (33, 34). Thus, it is thought that acute neuroinflammatory responses are beneficial in the CNS, since they halt additional injury and facilitate the survival of neurons (35).

In contrast to the response seen during acute neuronal injury, chronic microglial activation observed in progressive neurodegenerative disorders is morphologically and functionally distinct. Persistent reactive microgliosis has been seen in postmortem brains of patients in all progressive neurodegenerative disorders (4, 36).

While the brain was widely regarded as an immune-privileged site where inflammation only occurred in the context of direct infection or BBB breakdown, it has now been proven that all endogenous CNS cell types express specialized pattern recognition receptors (PRRs) that can trigger an innate immune response to specific host-derived molecules called danger/damage associated molecular pattern (DAMPs) (9, 37, 38). Endogenous DAMPs can directly induce neuroinflammation in the CNS, shifting immunocompetent cells from their beneficial roles to a chronically reactive state, thus contributing to the progression of neurodegeneration (9). This view has changed our understanding and therapeutic approach to neurodegenerative disorders from a neuron-centric perspective of preventing neuronal death or providing trophic support for degenerating neuronal populations. An important common aspect of most progressive neurodegenerative diseases is the presence of persistent insoluble protein aggregates. These aggregates may function as DAMPs to activate PRRs [Toll-like receptors (TLRs); nucleotide-binding domain (NOD) leucine-rich repeat (LRR), and pyrin domain–containing 3 (NLRP3); receptor for advanced glycation end products (RAGE), etc.] on microglia and, thus, they mediate chronic reactive gliosis and neuroinflammation.

Another finding commonly seen in neurodegenerative diseases is an exaggerated release of pro-inflammatory cytokines and chemokines in the CNS such as TNFα and the over expression of IL-1β (39–42). Numerous cytokines and chemokines of the TNF and IL superfamily are also increased in the cerebrospinal fluid (CSF) and serum of patients with neurodegenerative diseases, making them potential markers of disease onset, progression, or treatment efficacy. However, no selective biomarkers have been established for progressive neurodegenerative diseases to this date.

The complement system is an additional major player of the innate immune system that can have a pathogenic role in progressive neurodegenerative diseases (43–46). Specific complement factors and receptors are also up-regulated in the serum and on circulating immune cells of neurodegenerative disease patients, suggesting that complement activation and signaling could be an important link between the CNS and peripheral arms of innate immune system in neurodegenerative diseases (47).

## Materials and Methods

A literature search was conducted to investigate the existence of candidate immunotherapies for neurological disorders by using key terms to identify relevant articles on the subject. No date restrictions had been set for the articles to be retrieved from the search. The terms used for the search strategy were grouped into three broad categories based on key concept words “neurological disorders” and “immunotherapy.” Combinations of terms from each category were grouped together and joined with the terms from the other categories to be used in our database search.

Category 1: Alzheimer's disease, Parkinson's disease, Synucleinopathy Amyotrophic lateral sclerosis and Huntington's disease

Category 2: Active immunotherapy, Passive immunotherapy, inflammatory mediators, pattern recognition receptors (**PRRs**), pathogen-associated molecular patterns (**PAMPs**).

### Eligibility Criteria

Studies of interest consisted of human clinical trial results and results acquired from trials on animal models. The following electronic databases were used: PubMed, OVID Medline, Web of Science, and Google scholar. Articles that did not assess the efficacy of the immunotherapy compared to untreated controls were excluded. All study designs were included; except case series, case reports, reviews, short communications, and letters to the editors. Studies that recruited patients with multiple comorbidities were excluded. In addition, studies conducted in non-English languages were also excluded.

## Results: Therapeutic Strategies Targeting Neuroinflammation in The Management Of Neurodegenerative Diseases

### Targeting Inflammatory Mediators

#### TNFα

A major therapeutic strategy is to target the production of secreted and cell surface inflammatory mediators driving neuronal dysfunction and death. Inhibition of soluble TNFα via small-molecule inhibitors or by viral over-expression has proven to be efficient in several experimental models of neurodegenerative disorders, including AD and PD (48, 49). Yet, in a randomized double-blind phase II clinical trial, weekly subcutaneous injections of Etanercept (a TNF inhibitor) did not improve cognition, global function, or behavior in a small group of subjects with mild-to-moderate AD dementia (50). The presence of safe and effective TNFα inhibitors, currently used for systemic inflammatory diseases, renders them potential therapeutic candidates if tested at earlier disease stages or in combination with other anti-inflammatory therapeutics (48).

#### NLRP3 and Interleukins

Some populations of neurons were found to be susceptible to the chronic production of IL-1β in the CNS (Godoy et al., 2008). Viral over-expression of IL-1β at low levels leads to DA degeneration in the substantia nigra; furthermore, inhibition of NLRP3-driven IL-1β secretion is protective in transgenic AD models (51). Inhibition of the NLRP3 inflammasome can constitute an effective way to block downstream IL-1β production, which is thought to be a master regulator of pro-inflammatory responses in the CNS (52). Tested in a preclinical mouse model of multiple sclerosis, the first small-molecule inhibitor of the NLRP3 inflammasome (MCC950) demonstrated therapeutic efficacy (53). Endogenous ketone bodies were also found to strongly inhibit the NLRP3 inflammasome, suggesting that CNS permeable compounds, such as β-hydroxybutyrate, may have a therapeutic role for neuroinflammatory diseases (54). In experimental models of AD, IL-12, and IL-23 were associated with neutralizing antibodies capable of some therapeutic benefit when given systemically (55). Additional studies are needed to evaluate the efficacy of these mechanisms in other neurodegenerative diseases. As US FDA-approved IL-12 neutralizing drugs are currently used in the management of psoriasis, it may be worthwhile to evaluate these drugs in AD and other neurodegenerative diseases (56). Deposition of complement around plaques and degenerating neurons were also seen in several neurodegenerative disorders (57–59). In models of AD, ALS, and HD, pharmacological inhibition of the receptor for the terminal complement component, C5a, was found to limit disease pathology and progression (60).

Novel research aimed at attenuating neurodegenerative disease pathology examines possible anti-inflammatory and immunomodulatory roles of molecules such as granulocyte-macrophage colony-stimulating factor (GM-CSF), peroxisome proliferator-activated receptor gamma (PPAR-γ) and glucagon-like peptide 1 (GLP-1).

#### GM-CSF

GM-CSF is an immunomodulatory growth factor and cytokine that is deregulated in neurodegenerative diseases. In fact, GM-CSF elicits its effects on dendritic cells to control the induction and proliferation of regulatory T (Treg) cells, as well as inducing microglial proliferation, ultimately controlling microglial homeostasis and regulating inflammation (61–64). GM-CSF has exhibited an extensive neuroprotective potential in both preclinical and clinical studies in AD. Administration of GM-CSF therapy to AD mouse models successfully attenuates neuroinflammation and cognitive decline by rescuing hippocampal neuronal pathways and enhancing Aβ clearance by recruiting microglia to amyloid plaques (65, 66). Data from clinical trials shows significant cognitive and memory improvement in groups treated with the 127-amino-acid synthetic recombinant form of GM-CSF, Sargamostim (GM-CSF Leukine), when compared to controls. Moreover, phase 2 trials (NCT01409915) involving Sargamostim have deemed it safe and tolerable for all AD patients (67). Sargamostim's immunomodulatory and neuroprotective roles are also being investigated in PD. Preliminary results are promising, as preclinical and phase 1 trials (NCT01882010) have shown significant reduction in PD associated motor symptoms, and that GM-CSF rescues and protects nigrostriatal dopaminergic neurons via Treg induction (68, 69). Additionally, Sargamostim is safe and generally well-tolerated among patients, aside from mild injection site reactions (68).

The efficacy of GM-CSF remains controversial in treating ALS patients due to inconsistencies in clinical results, with some studies showing that GM-CSF does not benefit ALS patients and does not slow down disease progression (70, 71). Current preclinical data suggest that GM-CSF extends the lifespan of transgenic mouse models and has potent anti-inflammatory capabilities by downregulating TNFα and reducing microglial activation (72, 73). Moreover, GM-CSF is neuroprotective; hence, it promotes microglial migration and recruitment to damaged axon segments and preserves large myelinated axons (72, 73). Current clinical studies have yielded conflicting results as disease mechanisms remain to be clearly understood. Aside from being safe and tolerable, the efficacy of GM-CSF in attenuating motor symptoms and alleviating ALS pathology seems rather inconsistent (74, 75). In this regard, GM-CSF has been reported as a potent anti-inflammatory agent in ALS, as it upregulates anti-inflammatory cytokines such as IL-10 and reduces pro-inflammatory cytokines such as TNFβ, monocyte chemoattractant protein-1, interferon-γ, IL-7 and IL-17 (76, 77). Furthermore, Johannessen et al. (77) and Zhang et al. suggest (78) that GM-CSF could play a role in attenuating motor decline and enhancing patient survival. On the other hand, Amirzagar et al. (70)and Nefussy et al. (71) provide evidence that GM-CSF offers no improvement to ALS-related clinical outcomes, with Amirgazar et al. reporting aggravation of disease status and acceleration of disease course, especially in females, in response to GM-CSF treatment.

#### GLP-1 and PPAR- γ

Despite GLP-1 agonists and PPAR- γ agonists being primarily anti-diabetic drugs, recent findings indicate that these molecules execute neuroprotective and anti-inflammatory functions, which alleviate symptoms of AD and PD as well as rescuing Akt-1 and m-TOR, and insulin signaling in the brain (79–84).

GLP-1 agonists reduce proinflammatory cytokines and reduce disease burden in AD and PD mouse models. The mechanisms detailing the mode of action of these drugs and their pathways have been extensively reviewed (85–87). Nevertheless, recent results obtained from AD and PD clinical trials for GLP-1 agonist drugs have shown minor improvements in disease pathophysiology, if any (88–91). These results are still preliminary and therefore warrant further investigation before proper conclusions can be drawn.

Pre-clinically, PPAR- γ agonists have been shown to reduce neuroinflammation, Aβ-42 load, phosphorylated tau, and synaptophysin, ultimately enhancing spatial memory and motor function in AD mouse models (81–93). Furthermore, two promising PPAR-γ agonists currently under phase II clinical trials, T3D-959 (NCT04251182), and Pioglitazone (NCT00982202), have proven their safety and tolerability among patients (94, 95). Moreover, T3D-959 significantly enhanced cognition and insulin metabolism in AD patients (94). Pre-clinically, Pioglitazone has shown promising results in PD, where it has markedly reduced neuroinflammation and microglial proliferation in multiple PD models; but these results are yet to be validated in clinical trials (96–98).

### Targeting the Interaction Between PRRs and DAMPs

PRRs, such as RAGE, Mac1, and TLRs, constitute potential targets for treatment, given the view that DAMPs and misfolded proteins mediate neuroinflammation by interacting with multiple PRRs. Moreover, stimulating PRRs with neuronal DAMPs was found to be associated with the activation of downstream pro-inflammatory pathways, such as NOX2, iNOS, and TNFα (1). Thus, efficient targeting of PRRs involved in each neurodegenerative disease could be a possible strategy to decrease reactive gliosis and chronic self-perpetuating neuroinflammation and neurotoxicity. An inhibitor of RAGE, PF-04494700, showed promising results in pre-clinical models, but results from clinical trials were inconclusive (99). Using transgenic AD models, NLRP3 inhibitors, such as MCC950, showed promising results, providing evidence that NLRP3 is an important intracellular PRR, which detects aggregated misfolded proteins (51).

### Vaccine Therapy

The mainstay in vaccine therapy consists of passive and active immunization with either monoclonal antibody infusion, or vaccination with specific antigens that induce adaptive immune responses, respectively. Passive immunization with monoclonal antibodies offers a reduction of the target molecules with a robust dosage management, but the drawbacks of this approach are high cost, frequent administration, and adverse side effects. In contrast, active immunization relies on the use of specific antigens, which induce production of antibodies or the modulation of inflammatory responses (100). Here, we summarize the status of vaccine therapy in neurodegenerative diseases.

### Synucleinopathies

#### Passive Immunization

The treatments for synucleinopathies aim to reduce α-syn accumulation and cell-to-cell transfer, and can be combined with drugs to reduce neuroinflammation, expecting synergistic effects.

##### Trials in Transgenic Mice

Passive immunization against synucleinopathies relies on different monoclonal antibodies against specific to different regions of α-syn. One such approach consists of using antibodies that target the N-terminal or the central region of α-syn. Rats with nigrostriatal degeneration were subjected to intraperitoneal administration at 2-week intervals with α-syn antibodies during a total 3-month period. Both types of antibodies exerted neuroprotective effects in terms of a reduction in α-syn-induced nigral cell death and a decrease in activated microglia in the substantia nigra, with the antibody targeting the α-syn N-terminal being the most efficient (101). Recently, a new monoclonal antibody, mAb47, has been developed against the protofibril structures of α-syn. This antibody could decrease the intracellular oligomerization of α-syn *in vitro* (102), and decreased the levels of α-syn protofibrils in the spinal cord *in vivo* (103). In an attempt to develop a novel passive immunization technique, naturally occurring anti-α-syn autoantibodies, NAbs-α-syn, were purified and isolated from intravenous immune globulin (IVIG) of healthy individuals and used to immunize A53T transgenic PD mouse model. NAbs-α-syn significantly reduced the levels of soluble α-syn, α-syn oligomers, and intracellular phosphorylated α-syn deposits. Ultimately, NAbs-α-syn successfully attenuated memory and motor deficits and neuroinflammation associated with PD (104).

##### Clinical Trials

###### Prasinezumab.

Prasinezumab, also known as PRX002, a C terminus targeting monoclonal antibody is currently under phase II clinical study, in which targeting α-syn aggregates was shown to significantly reduce free serum α-syn, but without any significant reduction of free CSF α-syn (105, 106). Preclinical results from the mouse monoclonal antibody 9E4, the precursor of PRX002, showed that internalization of the antibody-α-syn aggregates complex occurs via the Fc-γ receptors on the surface of microglial cells, leading to a reduction in α-syn concentration (106). Results of the phase I clinical trial showed that PRX002 antibody levels increased in patient CSF in a dose-dependent manner, which might indicate that PRX002 might target α-syn extracellular aggregates in the CNS (105). PRX002 was shown to be safe and well-tolerated, and does not cause immunogenicity in the host (105, 107).

###### BIIB054.

BIIB054 is an N-terminus targeting monoclonal antibody currently under phase II clinical study that preferentially targets α-syn aggregates (108). The current study showed that BIIB054 formed complexes with serum α-syn and described it as a safe and tolerable candidate monoclonal antibody against α-syn (109).

#### Active Immunization

Mice vaccinated with recombinant human α-syn produced high affinity α-syn antibodies mainly targeting the C-terminus of α-syn (110). Interestingly, the immunized mice showed reduced accumulation of α-syn in neuronal cell bodies and synapses in the temporal cortex and a higher conservation of synaptophysin-positive nerve terminals (110). It was proposed that α-syn is degraded by lysosomal processing. Immunization against α-syn has also been explored in a rat model of PD based on the viral delivery of human α-syn into the nigrostriatal pathway. Interestingly, this form of active immunization resulted in an adaptive immune response mediated by CD4–positive, Foxp3–positive cells that successfully infiltrated the nigrostriatal system. As a result, decreased α-syn inclusions in the substantia nigra were observed in the experimental group, indicating that the observed adaptive immune responses possess therapeutic potential (111). Moreover, AFFITOPE consists of active vaccination with short α-syn peptides, which has provided promising results *in-vivo*. AFFITOPE successfully generated anti-α-syn antibodies that infiltrated the CSF and plasma in high titres, targeted α-syn aggregates, and reduced neuronal degeneration by attenuating oligomeric α-syn accumulation in neurons (112–114). AFFITOPE is currently undergoing extensive phase I clinical trials that are yielding promising results displaying its safety and tolerability among patients (114).

Overall, the outcomes from preclinical studies investigating both active and passive immunization strategies in several models of α-synucleinopathies are considered as promising.

### Amyloid β and Tau

#### Passive Immunization

Passive immunization against AD is currently the most advanced immunotherapy under development.

##### Amyloid β Targeting Therapies

###### Trials in Transgenic Mice.

An anti-Aβ antibody targeting the Aβ 31–35 sequence showed positive immunological results and reduction of disease pathology in transgenic mice. The antibody effectively bound to Aβ-42 species and attenuated their toxicity and prevented Aβ-42 induced cell death as well as restoring hippocampal synaptic plasticity. Furthermore, anti Aβ31–35 antibody rescued spatial learning and memory, which makes targeting the Aβ31–35 sequence a promising candidate for future investigation in the quest for passive immunization therapies for AD (115).

##### Clinical Trials

###### Bapineuzumab.

The first studied candidate monoclonal antibody was bapineuzumab, which targets the Aβ N-terminus and possesses a higher affinity for deposited amyloid plaques than soluble Aβ monomers (116, 117). However, multiple studies and a meta-analysis conducted by Abushouk et al. assessing the potential use of bapineuzumab in the treatment of AD, reported significant association with severe adverse events and no enhancement of cognitive decline, and therefore concluded that bapineuzumab is not recommended for use in the treatment of AD and clinical trials have since then been discontinued (117–123).

###### Solaneuzumab.

Solanezumab is a monoclonal antibody that targets the mid domain of the Aβ peptide and selectively binds to monomeric, soluble Aβ (123–126). However, due to its failure in reducing Aβ burden and in light of recent shortcomings in phase III trials that revealed that solaneuzumab did not improve cognitive decline in patients with mild AD, current trials were terminated due to less and less promising results (127, 128).

###### Gantenerumab.

Gantenerumab is a monoclonal antibody that targets aggregated Aβ in the brain. It targets the N terminus as well as the mid-domain of the Aβ peptide, and shows high affinity for aggregated amyloid β species. The evaluation of this candidate in a phase III clinical trial indicated a dosage–dependent reduction of Aβ plaques in the brain below threshold for healthy levels (129, 130). Moreover, it has been shown to ameliorate mental status and decrease cognitive decline in patients, especially in early stages of the disease (130, 131).

###### Crenezumab.

Crenezumab is a monoclonal antibody targeting the mid-domain of Aβ with reduced effector function, and it binds to Aβ oligomers, fibrils, and plaques, limiting aggregation and facilitating disaggregation (132). One advantage of this antibody is that it is an IgG4 antibody, which shows reduced pro-inflammatory activity and limited risk of vasogenic edema (132–134). Despite evidence of tolerability of Crenezumab among patients and Aβ plaque reduction (135), overall cognitive decline and disease pathology were not improved, and therefore the efficacy of the treatment remains in question (136, 137). After launching a phase III investigation for Crenenzumab's potential use as treatment or preventive therapy for patients suffering from familial AD (138), an interim analysis, showed that it was unlikely to meet primary endpoints. Clinical trials involving Crenezumab have been since then terminated.

###### Aducanumab.

Aducanumab is a monoclonal antibody that is currently under phase III testing. It can bind the N-terminal of both soluble and insoluble Aβ species, with recent evidence emphasizing its strict high affinity binding to aggregated and pathogenic forms of Aβ (139). Studies conducted on aducanumab collectively agree on the tolerability of the antibody and report minimal treatment-induced adverse effects. In addition, aducanumab has been shown to infiltrate different brain regions and reduce Aβ plaques as well as slowing disease progression (140–142). Trials involving Aducanumab have been since then terminated due to futility of analysis.

###### Ponezumab.

Ponezumab is a monoclonal antibody that targets soluble amyloid beta by binding to the C-terminal of the sequence (143). It is speculated to reduce CNS amyloid beta species by sequestration of free blood Aβ and therefore shifting the blood-brain Aβ equilibrium toward the blood (144, 145). In the study conducted by Landen et al., the tolerability and effectiveness of treatment with ponezumanb was assessed. It was found that ponezumab had a reduced likelihood of inducing adverse events and was therefore tolerated in patients of the study group. On the other hand, ponezumab showed no effect on cognitive abilities in patients and did not reduce the severity of disease pathology (146–148). Notably, ponezumab showed an increase of free Aβ species in the blood without infiltrating the CSF. This increase was explained by the mode of action of ponezumab, which was discussed above (147). Based on the above, ponezumab clinical trials have been discontinued.

##### Tau Targeting Therapies

###### Trials on Transgenic Mice.

*CBTAU-22.1* In a novel approach, and in an effort to enhance the therapeutic potential of a naturally occurring tau specific human antibody CBTAU-22.1, van Ameijde et al. successfully improved the affinity of CBTAU-22.1 through random mutagenesis and a variant, dmCBTAU-22.1, was generated. *In vitro*, dmCBTAU-22.1 reduced pathological tau aggregation, seeding and spread. *In vivo*, P301L transgenic mice were treated with dmCBTAU-22.1, which successfully reduced tau paired helical filaments as compared to controls (149).

###### 43D, 77E9, and Antibody D.

Mouse monoclonal antibody 43D, which targets the N terminal projection of tau, produced the most pronounced results of the three. Triple transgenic (3xTG) mice treated with antibody 43D exhibited reduced total tau, p-tau, p-tau seeding and propagation, as well as attenuation of the amyloid beta and amyloid precursor protein load in the hippocampus (150, 151). Moreover, antibody 77E9 exhibited similar effects as 43D in attenuating disease pathology, excluding the effects of 43D seen on p-tau and amyloid beta (151). Dai et al. evaluated the effect of a combined dose of 77E9 and 43D antibodies, but the results were less effective than the sole use of each antibody (151). Both antibodies reduced short-term and spatial memory impairments in 3xTG mice (151). The third of these antibodies, antibody D, is a tau antibody that recognizes the central region of tau protein. In the context of passive immunization, antibody D reduced tau seeding and propagation of tau pathology to different brain regions (152).

###### Anti-Phosphorylated Tau Antibodies.

Passive immunization with specific monoclonal antibodies targeting phosphorylated tau is another promising approach to treat AD; however, their use is currently limited to test animals. D'Abramo investigated the use of three anti-phosphorylated tau antibodies: RZ3, CP13, and PG5 as antibody therapies for AD in JNLP3 transgenic mice. Of these three antibodies, CP13, which targets phosphorylated Serine residue 202 of tau, was the only one to successfully reduce insoluble or soluble tau species in the cortex and hindbrain of transgenic mice (153).

###### TOMA.

Gerson et al. was the only team that investigated the use of anti-tau oligomer antibodies in treatment of PD. Using A53T transgenic mice overexpressing mutated α-synuclein, they found that the use of tau oligomer-specific monoclonal antibody (TOMA) significantly reduced toxic tau oligomers, which in turn reduced α-synuclein oligomers with fibril-like characteristics as well as reducing Lewy Body structures. Ultimately, TOMA protected the immunized mice from cognitive and motor deficits related to PD (154). Moreover, TOMA was also tested on different AD mouse models, JNLP3, Htau mice and Tg2576, and yielded promising results. TOMA successfully targeted toxic oligomeric tau species, reduced memory deficits and cognitive decline, ultimately slowing down disease progression (155–157).

##### Clinical Trials

Monoclonal antibodies targeting tau protein have started to make the leap from animal testing to clinical trials. From these, C2N 8E12, gosuranemab, zagotenemab, and semorinemab are worthy of mention, as they are currently being assessed for clinical tolerability in phase II trials. Preliminary safety and tolerability results are promising thus far; however, information on these treatments remains rudimentary at best, hence further research is required to evaluate their clinical efficacy and safety (158–162).

### Active Immunization

#### Amyloid β Targeting Therapies

##### Amyloid β Targeting Therapies.

AN-1792 The first vaccine tested in humans (starting in 2000), called AN-1792, consisted of a full-length pre-aggregated amyloid peptide (Aβ1–42). AN-1792 successfully cleared Aβ40, Aβ42 and Aβ43 from AD plaques. This vaccine, however, led to severe side effects, including aseptic meningoencephalitis, in ~6% of AD patients (100, 163–167). Due to these side effects, AN-1792 trials have since been terminated.

##### ACI-24.

ACI-24 is a liposome-based vaccine designed to induce an antibody response against aggregated Aβ peptides. In preclinical studies, repeated subcutaneous injection of ACI-24 into AD transgenic mice yielded high titers of anti-Aβ antibodies, reducing the concentration of insoluble Aβ1-40, and Aβ1-42 (168). Preliminary phase I results are promising, with ACI-24 being well-tolerated among patients, additionally, the vaccine has successfully triggered an immune response in patients (169). ACI-24 is currently under phase II clinical trial for safety, tolerability and immunogenicity for its use on patients with AD.

##### CAD 106.

CAD106 is an anti-Aβ vaccine generated from multiple copies of the Aβ1–6 peptide. In a phase II study, CAD106 was well-tolerated in the study group with a minimal number of treatment related adverse effects, which did not negatively affect treatment tolerability and safety. CAD106 successfully increased anti-Aβ antibody titers, which successfully targeted and cleared Aβ species. Moreover, the treatment did not cause occurrences of meningoencephalitis, autoimmune disease or CNS inflammation (170–172).

##### ACC-001.

ACC-001, constituted of N terminal (Aβ1-7) peptide, is another vaccine that has successfully undergone phase II trials. ACC-001 was shown to be well-tolerated by patients enrolled in the study, and to induce a robust immune response mediated by anti-Aβ IgG. Coupling ACC-001 with QS-1 as an adjuvant further increased the robustness of the observed immune response (173–176).

##### ABvac40.

ABvac40 is a vaccine formulated from repeats generated from C-terminus fragments of Aβ40. It has successfully generated specific anti-Aβ_40_ antibodies in patients with mild to moderate AD. Initial tolerability and safety tests have been successful, with minor injection site reactions and adverse events being recorded; moreover, treatment did not trigger vasogenic edema or microhemorrhage. Based on the promising phase I results, ABvac40 has moved to phase II clinical trial testing in which its efficacy and impact on cognition will be assessed (177).

#### Tau Targeting Therapies

##### Trials in Transgenic Mice.

Tau379–408[P-Ser396, 404] In a different approach, the effectiveness of phosphorylated tau peptide, Tau379–408[P-Ser396, 404], as an immunogen was tested in 3xTG mice. This study demonstrated that phosphorylated tau peptide vaccine triggered the generation of anti-tau antibodies which led to reduction of pathological tau aggregates, clearance of both soluble and insoluble tau species, and the reduction of Aβ deposition (178). These preliminary results show the potential use of tau peptide in active immunization against AD pathology and the need to assess the potential clinical application of these vaccines through clinical trials.

#### Clinical Trials

##### AADvac1.

Tau active immunization approaches represent another path under exploration. AADvac1 is a new vaccine that can specifically recognize pathological tau oligomers. Active immunotherapy with this vaccine decreased the extent of oligomers and of neurofibrillary pathology in the brains of transgenic rats (14). The formulation comprises a synthetic peptide from the tau aggregation domain. AADvac1 successfully triggered anti-tau antibody production, and adverse effects were limited to injection site reactions. Moreover, preliminary data indicated a reduction in cognitive decline in patients undergoing the treatment. A phase II clinical trial involving AADvac1 has been launched to assess its efficacy (179–181).

##### ACI-35.

ACI-35 is a liposomal-based vaccine consisting of a synthetic tau peptide phosphorylated on pathological residues S396 and S404 thus resembling the phospho-epitope of tau. The evaluation in test animals, wild-type C57BL/6, and P301L, indicated a positive immunogenic activity specifically targeting pathological phosphorylated tau species with no neurological side effects or inflammation of neural tissue (182). Preliminary findings from phase I trial indicated that the treatment is tolerable and safe, but was not efficiently immunogenic; therefore, a second-generation vaccine, ACI-35.030, has been generated. ACI-35.030 is 5 times more immunogenic than its predecessor and will replace ACI-35 in the phase I/II safety and tolerability clinical trial (183).

### Superoxide Dismutase 1

#### Active Immunization

Superoxide dismutase 1 (SOD1), an enzyme known for its role in relieving oxidative stress, has been studied thoroughly as a target for mutations in ALS. The cause of 20% of familial ALS cases has been attributed to mutated SOD1 (184). Currently, there is no consensus on mutant SOD1's pathological function in ALS, but evidence suggests that mutant SOD1 might interfere with cellular metabolism (185–189) and oxidative stress pathways (190), and might cause metal dyshomeostsis (191–194). Takeuchi et al. have investigated the efficiency of a vaccine targeting extracellular SOD1 in immunizing transgenic mice against ALS (195). In their experiment, they tested the efficiency of mutant SOD1 (G93A mutant SOD1) and wild-type SOD1 (wt-SOD1) vaccines in relieving the symptoms and reducing the pathogenicity of ALS. Takeuchi et al. generated the vaccine based on these two types of SOD1 due to the diversity of mutations that typically affect this enzyme in ALS (196–198). Upon inoculation, wt-SOD1 vaccine extended life expectancy and delayed disease onset, while both wt-SOD1 and mutant SOD1 vaccinations reduced anterior horn motor neuron loss (195). A similar vaccine was modeled based on mutant SOD1 and tested on G37R SOD1 and G93A SOD1 transgenic mice and resulted in significant life span extension and delay in disease onset in mice that do not suffer from an overexpression of the mutant SOD1 phenotype, i.e. G37R SOD1 (199).

#### Passive Immunization

Antibody therapy has been investigated against SOD1 mutant oligomers. Passive immunization of SOD1-G93A mice with anti-SOD1 antibody (W20) targeting toxic soluble SOD1 oligomers protected motor neurons from apoptosis and extended their survival. This was achieved by reducing neurotoxic SOD1 oligomer aggregates and substantially inhibiting gliosis and neuroinflammation in the spinal cords and brain stems of transgenic mice. Ultimately, W20 improved motor neuron survival and motor performance (200).

### C9orf72

#### Passive Immunization

C9orf72 is a protein coding gene involved in endosomal trafficking (201). Mutation of the hexanucleotidic GGGGCC (G4C2) intronic repeat, upstream the C9orf72 coding sequence, leads to the expansion of this repeat from 2 to 22 copies to 700–1,600 copies (202, 203). The G4C2 expansion can be translated by repeat-associated non-ATG (RAN) translation to generate dipeptide repeat protein aggregates (DPR): poly GA, poly GP, poly GR, poly PA, and poly PR (204, 205). Poly GA has been described as an abundant and cytotoxic DPR in brain and spinal cord neuronal inclusions in ALS patients (205–207). The exact role of Poly GA and its contribution to disease pathology remains under study. It was shown to act as a nucleation seed to promote DPR aggregation, it enhanced G4C2 repeat expression and translation, and it could be transmitted between neurons (Qihui 207). This indicates that poly GA potentially aids in the spread of disease pathology in affected individuals. Poly GA causes reduced dendritic branching, proteasomal inhibition, apoptosis, and endoplasmic reticulum stress (206, 207). Zhou et al. investigated poly GA as a candidate target for immunotherapy for ALS. Treatment of poly GA transfected cells with anti-GA antibody showed a significant decrease in poly-GA seeding, aggregation and spread, suggesting the potential application of anti-poly GA antibodies as immunotherapies for ALS (208). Recently, Nguyen et al. investigated the potential use of anti-GA RAN antibodies in treating Frontotemporal Dementia (FTD) and ALS. Herein, passive immunization of FTD/ALS C9-BAC transgenic mice with anti-GA RAN antibodies, α-GA_1_, α-GP_1_, and α-GA_2_, has effectively reduced neuroinflammation and neurodegeneration in the anterior and posterior horns of the lumbar spine (209). Moreover, anti-GA RAN antibodies successfully rescued proteasome activity and subsequently reduced pathogenic poly-GA aggregates. Collectively, these changes significantly reduced physical and behavioral pathology, as well as survival, thus anti-GA RAN antibodies qualify as promising candidate therapies for ALS and FTD that merit further assessment in clinical trials (209).

#### Active Immunization

Novel findings indicate the possible use of poly-GA coupled with ovalbumin, OVA-(GA)_10_, as an active immunization agent. OVA-(GA)_10_ successfully elicited a robust immune response and induced an increase in anti-GA antibody titres in transgenic GA-CFP mice (210). Moreover, the produced anti-GA antibodies exhibited a high specificity to poly-GA aggregates resulting in their reduction in the brains of transgenic mice, ultimately reducing motor deficits and neurodegeneration (210). OVA-(GA)_10_ also attenuated neuroinflammation by normalizing the number of activated microglial cells and macrophages and reversing TDP-43 cytoplasmic mis-localization in the spinal cord. These findings corroborate the possible application of OVA-(GA)_10_ as a candidate vaccine to reduce disease severity and pathology in ALS and FTD (210).

### Mutant Huntingtin Protein

#### Active Immunization

Current reports on active and passive immunization against mutant Huntingtin (m-Htt) proteins remain inconclusive. Results obtained for active immunization are preliminary. A study conducted on a candidate plasmid vaccine administered to HDR6/2 mice proved to have no effect on the number of m-Htt aggregates (211). Another study examined the use of peptide, protein, and DNA plasmid vaccines against m-Htt and detected a robust antibody production after vaccination with a combination of three non-overlapping HTT exon1 peptides. However, this study did not assess the efficacy of the developed vaccine in reducing m-Htt aggregates (212).

## Discussion

**Figure 1:**
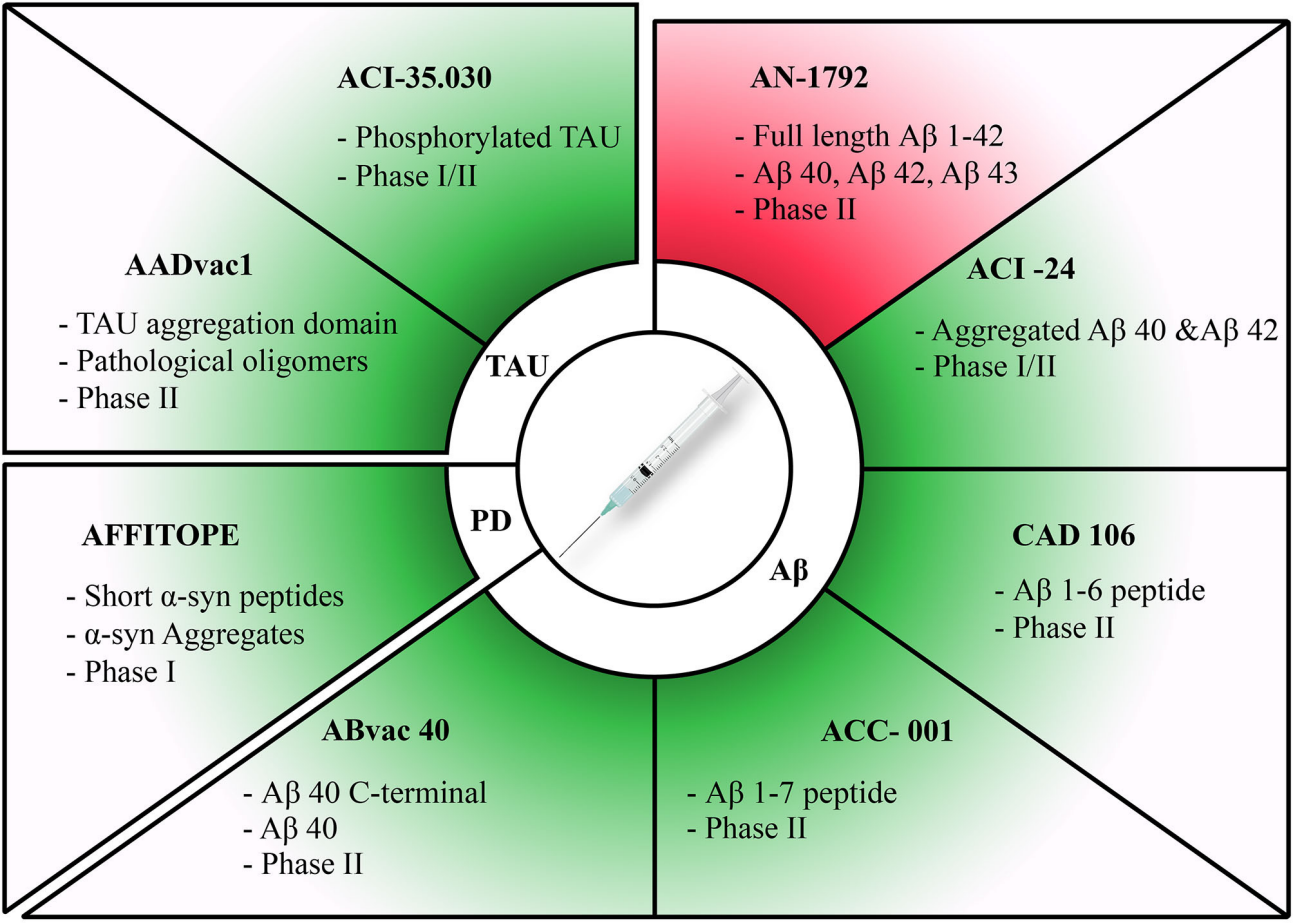
Summary of active immunization therapies assessed in clinical trials for different diseases. Discontinued trials highlighted in red and ongoing trials highlighted in green. Therapies have been categorized according to each pathology they target. The target of each therapy along with its current clinical trial phase have been stated for each therapy.

Emerging evidence supports the use of immunotherapeutic agents in the management of neurodegenerative diseases. Our understanding of immunomodulatory mechanisms in the CNS has greatly evolved in recent years. In this work, we discussed the pathogenesis of neuroinflammation and its role in neurodegeneration. The switch from a succinct limited immune response to a sustained chronic response appears to be the initiating event in disease onset. Understanding the exact mechanism behind this switch is essential to halt its progression and prevent occurrence of disease. Increased release of cytokines and chemokines by overactive immune cells further exacerbates the damage and accentuates neurodegeneration. We then discussed immunotherapeutic modalities that could revolutionize the management of so far intractable neurodegenerative diseases, with a focus on passive and active therapies targeting hallmark disease biomarkers due to the availability of extensive reviews covering neuroinflammatory targets. Vaccination therapy is an interesting approach as vaccines are considered main players in preventive medicine and public health (Figure 1, Table 1). More research on the pathophysiology and structures of Aβ, tau, and α-syn will bring us a step forward toward tailoring stronger vaccines capable of mounting specific immune responses against the accumulation of these proteins. Of major importance is the avoidance of serious secondary adverse events such as meningoencephalitis or possibly the induction of seizures upon introducing the abnormal protein aggregates. In this regard, Aβ42-specific T-cell activation has been demonstrated to cause multiple undesirable effects in AD patients including meningoencephalitis which could potentially exacerbate patient health status (213, 214). Since Aβ active immunization introduces Aβ peptides into the blood stream, these could serve as an immunogenic activator of an anti-Aβ T-cell mediated immune response. A suggested mechanism to bypass this undesirable response was to design a chimeric Aβ vaccine where Aβ immunogen presentation is carried out by Norovirus particles. This approach significantly enhanced immunogenicity and antibody production without Aβ42-specific T-cell activation (215, 216). Another suggested mechanism to bypass Aβ42-specific T-cell activation was by using peptide fragments as immunogenic particles for active immunization against AD instead of full-length peptides (171, 173). These findings further substantiate the necessity for future investigation to focus on the type of immunogenic particle, mode of delivery, and antigenic presentation of the immunogen by the vaccine.

**Table 1:** Study characteristics of active immunotherapy clinical trials.

**References**	**Therapeutic molecule**	**Disease**	**Target**	**Latest trial identifier**	**Results to date**
McFarthing and Simuni (114)	AFFITOPE	Parkinson's disease	α-syn	Phase I: NCT01568099	Safe and well-tolerated Immunogenic
Bayer et al. (164), Nicoll et al. (165), Nicoll et al. (166), Sakai et al. (167)	AN-1792	Alzheimer's disease	Full-length pre-aggregated Aβ	Terminated	Not safe Aβ40, Aβ42, and Aβ43 clearance
Immune (169)	ACI-24	Alzheimer's disease	Aggregated Aβ peptides	Phase II: 2018-000445-39	Safe and tolerated
Farlow et al. (170), Vandenberghe et al. (171), Winblad et al. (172)	CAD 106	Alzheimer's disease	Aβ1–6 peptide	Phase II: NCT01023685 NCT00795418	Safe and tolerated Production of anti-Aβ antibodies
Hull et al. (173), Ketter et al. (174), Pasquier et al. (175), van Dyck et al. (176)	ACC-001	Alzheimer's disease	N terminal (Aβ1-7) peptide	Phase II: NCT00479557 NCT01227564 NCT01284387	Safe and tolerated Production of anti-Aβ antibodies
Lacosta et al. (177)	ABvac40	Alzheimer's disease	C-terminal fragments of Aβ40	Phase II: NCT03461276	Safe and tolerable Increase in anti-Aβ40 antibodies
Kontsekova et al. (179), Novak et al. (180), Novak et al. (181)	AADvac1	Alzheimer's disease	Synthetic tau aggregation domain peptide	Phase II: NCT02579252	Safe and tolerable Triggers anti-tau antibody production Reduction of cognitive decline
Ayalon et al. (183)	ACI-35	Alzheimer's disease	Synthetic tau S396 –S404 phospho-epitope	Phase II: NCT04445831	Safe and tolerated Mild anti-tau antibodies

**Figure 2:**
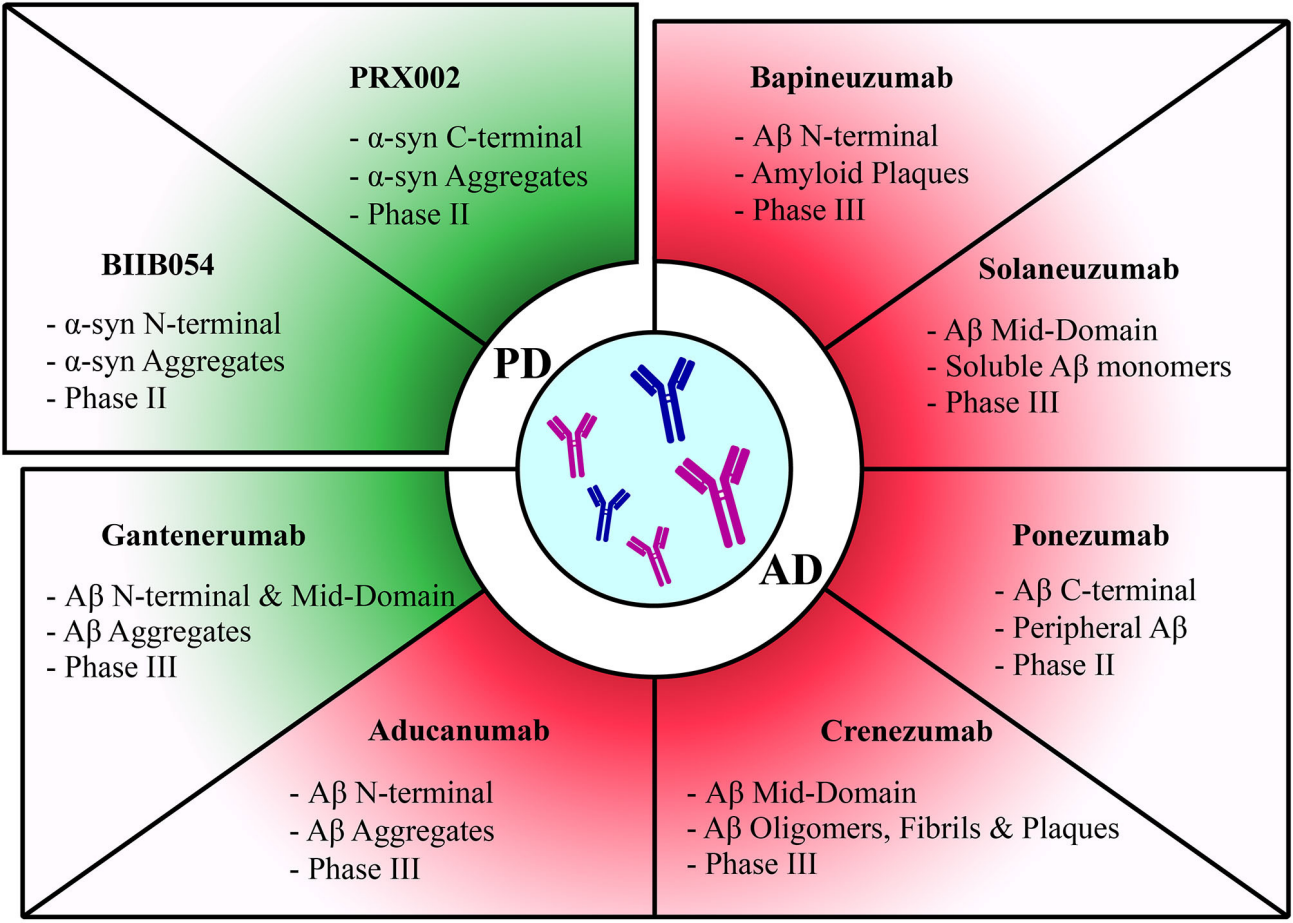
Summary of passive immunization therapies assessed in clinical trials for different diseases. Discontinued trials highlighted in red and ongoing trials highlighted in green. Therapies have been categorized according to each pathology they target. The target of each therapy along with its current clinical trial phase have been stated for each therapy.

The mode of administration of the vaccine represents another important consideration for ameliorating efficacy. Recently, there has been a focus on passive immunization with monoclonal antibodies, which may represent a safer approach than active immunization (Figure 2, Table 2). Overall, therapeutic antibodies are one of the fastest growing areas in the pharmaceutical industry for the treatment of cancer, autoimmune disorders, and now also for neurodegenerative disorders. Furthermore, another pivotal factor related to peptide based vaccines is the choice of the carrier protein which has been shown to impact the duration and extent of the elicited humoral immune response (217, 218).

**Table 2:** Study characteristics of passive immunotherapy clinical trials.

**References**	**Therapeutic molecule**	**Disease**	**Target**	**Latest trial identifier**	**Outcome**
Jankovic et al. (105)	Prasinezumab (PRX002)	Parkinson's disease	α-syn C-terminal	Phase II: NCT03100149	Safe and well-tolerated
Brys et al. (109)	BIIB054	Parkinson's disease	α-syn N-terminal α-syn aggregates	Phase II: NCT03318523	Safe and well-tolerated
Abushouk et al. (118), Bard et al. (119), Farlow and Brosch (120), Lu and Brashear (121), Salloway et al. (117), Salloway et al. (122), Siemers et al. (123)	Bapinezumab	Alzheimer's disease	Aβ N-terminal	Terminated	Severe adverse events No effect on cognitive decline
Doggrell (127), Honig et al. (128)	Solanezumab	Alzheimer's disease	Aβ mid domain Monomeric Aβ	Terminated	Does not reduce Aβ plaques No effect on cognitive decline
Klein et al. (130), Ostrowitzki et al. (131), Jia et al. (129)	Gantenerumab	Alzheimer's disease	Aβ N-terminal Aβ mid domain Aβ aggregates	Phase III: NCT01224106	Reduction in Aβ plaques Decrease in cognitive decline
Cummings et al. (136), Salloway et al. (137), Tariot et al. (138)	Crenezumab	Alzheimer's disease	Aβ mid domain Aβ oligomers Aβ fibrils Aβ plaques	Phase III: NCT03491150	Safe and tolerable Reduction in Aβ plaques No effect on cognitive decline
Budd et al. (140), Ferrero et al. (141), Sevigny et al. (142)	Aducanumab	Alzheimer's disease	Aβ N-terminal Aβ soluble Aβ insoluble Aβ aggregates	Phase III: NCT02477800 and NCT02484547	Safe and tolerable Reduction in Aβ plaques
Landen et al. (146), Landen et al. (147), Landen et al. (148)	Ponezumab	Alzheimer's disease	Aβ C-terminal Aβ soluble	Terminated	Safe and tolerable No effect on cognitive decline No effect on disease severity

The search for novel targets for the administered passive and active immunization therapies might provide new strategies for treatment. In a novel approach, Thomas et al. examined the possibility of indirectly targeting dysfunctional tau peptide by passively immunizing transgenic mice against BIN1 (bridging integrator 1) gene product (219). The gene BIN1 has been associated with AD, and its product, Myc box-dependent-interacting protein 1, has been shown to co-localize and interact with tau and enhance its pathogenicity by promoting tau release and neurotoxicity (220–223). Anti-BIN1 antibody reduced p-tau species and increased survival in P301S transgenic mice (219). In light of these findings, immunotherapeutic strategies might benefit from exploring novel targets to indirectly reduce disease pathology rather than exclusively target conventional disease biomarkers, which have so far provided limited promise and success.

Understanding the different steps of neuroinflammation provided useful insights into possible options to break the chain of events leading to disease. Modulating the innate immune system remains a plausible approach in managing neurodegeneration, similarly to several other autoimmune and inflammatory diseases. Nevertheless, new approaches currently under investigation could become pivotal therapeutic options in the future. Recent reports suggested that microglial modulation by the gut microbiota can be an exciting novel therapeutic target (224). In fact, PD patients were found to have an altered gut microbiota compared to matched healthy cohorts (225). Furthermore, dysregulation in sleep patterns is seen in several neurodegenerative diseases, and recent data support the view that altered sleep can affect neuroinflammation (226). The exact mechanisms by which dysregulated sleep modulates pro- or anti-inflammatory responses in the CNS are not fully understood yet. Lastly, the effects of systemic immune responses in modulating acute and chronic neuroinflammation are being elucidated. The recently discovered lymphatic vessels in the brain which can transport fluid and immune cells from the CSF constitute another relevant finding (227). These CNS lymphatic networks interconnected to deep cervical lymph nodes could play a role as peripheral immune mediators in regulating neuroinflammatory responses.

The burden of neurological disease is growing globally, and neurodegenerative disorders represent major unmet medical needs and costs to healthcare systems worldwide. The challenges of translating scientific advances into new therapies in neurology are increasing, which are partly due to the lack of patients' willingness to participate in clinical trials and the complexity of developing neurotherapeutics due to a paucity of validated biomarkers (228), longer duration of clinical trials, and higher failure rates due to lack of efficacy (229). Examples of factors determining the ability of a therapeutic candidate to be considered as a potential successful drug include efficient delivery of this candidate, with the appropriate dosage to act on the intended cell or tissue for a specific duration of time; obviously when the target is within the CNS, difficulties and uncertainties arise due the complexed nature of the brain, the intricacies of models mimicking human neurological diseases and the poor functional outcome measures (230). Nevertheless, in searching for more efficient treatments, immunotherapy targeting abnormal protein aggregates or inflammatory molecules is emerging as a promising therapeutic strategy. Our basic understanding of innate immune responses in the CNS during healthy aging and in neurodegenerative diseases has largely progressed in the last two decades. Yet, significant knowledge gaps still exist in understanding the full mechanisms of beneficial and pathologic neuroinflammatory responses tilting the balance toward healthy or diseased aging. With the emergence of additional insights into neuron–glial and glial–glial interactions in the CNS, targeted and potentially more effective therapeutic strategies can be attained. So far, late intervention seems to be the most important cause for treatment failure in several trials, meaning that to battle these diseases, the treatment essentially needs to be started at an early stage. Thus, in addition to the development of more efficacious drugs, better diagnostic strategies are warranted to diagnose these disorders at a time when there still has been no or only limited damage to the CNS. Recent research efforts surrounding neurological diseases are directed toward discovering valid disease biomarkers from body fluids; and good candidates (other than blood and CSF) offering promise as a biomarker pool for neurological disease diagnosis and monitoring, are urine and saliva (231). Finally, limitations in our understanding of the interplay between the innate response in the CNS and systemic immunity are challenges that need to be overcome. The innate immune system, therefore, provides exciting opportunities for disease-modifying treatments in the CNS that are both innovative and feasible. As our knowledge of the precise underling immune mechanisms advances, more effective therapies will be developed in managing these so far intractable neurodegenerative diseases.

## Author Contributions

All authors listed have made a substantial, direct and intellectual contribution to the work, and approved it for publication.

## Conflict of Interest

The authors declare that the research was conducted in the absence of any commercial or financial relationships that could be construed as a potential conflict of interest.
